# The Global Workspace (GW) Theory of Consciousness and Epilepsy

**DOI:** 10.3233/BEN-2011-0313

**Published:** 2011-03-29

**Authors:** Fabrice Bartolomei, Lionel Naccache

**Affiliations:** ^1^INSERMU751Laboratoire de Neurophysiologie et NeuropsychologieMarseilleF-13005France; ^2^Université de la MéditerranéeFaculté de MédecineMarseilleF-13005 France; ^3^CHU TimoneService de Neurophysiologie CliniqueAssistance Publique des Hôpitaux de MarseilleMarseilleF-13005France; ^4^AP-HPGroupe hospitalier Pitié-SalpêtrièreDepartments of Neurophysiology & NeurologyParisFrance; ^5^INSERMICM Research CenterUMRS 975ParisFrance; ^6^Université Paris 6Faculté de Médecine Pitié-SalpêtrièreParisFrance

**Keywords:** Consciousness, temporal lobe epilepsy, global workspace, synchrony

## Abstract

The global workspace (GW) theory proposes that conscious processing results from coherent neuronal activity between widely distributed brain regions, with fronto-parietal associative cortices as key elements. In this model, transition between conscious and non conscious states are predicted to be caused by abrupt non-linear massive changes of the level of coherence within this distributed neural space. Epileptic seizures offer a unique model to explore the validity of this central hypothesis. Seizures are often characterized by the occurrence of brutal alterations of consciousness (AOC) which are largely negatively impacting patients' lives. Recently, we have shown that these sudden AOC are contemporary to non-linear increases of neural synchrony within distant cortico-cortical and cortico-thalamic networks. We interpreted these results in the light of GW theory, and suggested that excessive synchrony could prevent this distributed network to reach the minimal level of differentiation and complexity necessary to the coding of conscious representations. These observations both confirm some predictions of the GW model, and further specify the physiological window of neural coherence (minimum and maximum) associated with conscious processing.

## 1. Introduction

Epilepsy is one of the most frequent brain disease affecting near 1% of the general population and negatively impacting quality of life [28].

There is a classical and operative distinction between generalized epilepsies in which the epileptic process affect bilaterally and widely the cerebral cortex and partial (or focal) seizures in which the seizures start from a limited part of the cerebral cortex and secondarily spread to other cortical and subcortical regions. Alteration of consciousness (AOC) is usual in generalized seizures and frequently occur in partial seizures. In this review we will focus on the mechanisms leading to AOC in partial seizures, and particularly in the most frequent type of chronic drug resistant partial epilepsies, the temporal lobe epilepsies (TLE).

AOC is the most dramatic clinical manifestation of TLE, causing important handicap and potential source of injury. In line with this, the international classification of epileptic seizures has made impaired consciousness the cornerstone by which the main categories of partial seizures, simple and complex, are distinguished [12]. It emphasizes an essential characteristic that in many cases has significant repercussions on the quality of life of the epileptic patient.

Whereas the structural and functional changes observed in TLE have been largely studied, the mechanisms leading to AOC are poorly known [8]. Several

ISSN 0953-4180/11/$27.50 © 2011 – IOS Press and the authors. All rights reserved theories/studies have proposed scenario about AOC in TLE (see below).

Recently we have proposed that AOC in temporal lobe seizures could be explained by an alteration of the "global workspace" functioning [3,13,17], a theory that we will developed in the next paragraphs.

## 2. The GW model of consciousness

The GW model, initiated by Bernard Baars' theoretical work [2,3] proposes that at any given time many modular cerebral networks are active in parallel and process information in an unconscious manner, while consciousness would correspond to the broadcasting of information to a global workspace. Dehaene, Changeux, Naccache and colleagues developed these principles, and elaborated a plausible functional neural architecture to the GW [15–17]. In particular they proposed that conscious access would be causally related to a mechanism of top-down attentional amplification into a self-sustained brain-scale state of coherent activity that involves many neurons distributed throughout the brain. The long-distance connectivity of these "workspace neurons" can, when they are active for a minimal duration, make the information available to a variety of processes including perceptual categorization, long-term memorization, evaluation, and intentional action. According to this model, global availability of information through the workspace is what we subjectively experience as a conscious state. Neurophysiological, anatomical, and brain-imaging data strongly argue for a major role of prefrontal cortex, anterior cingulate, and the areas that connect to them, in creating the postulated brain-scale workspace.

Importantly, two aspects of consciousness have to be distinguished. On the one hand 'access consciousness' refers to the neural mechanisms enabling an initially non-conscious perceptual representation, encoded in a specialized local cortical processor, to be broadcasted to the GW network as a conscious percept. In other terms, the issue of 'access consciousness' explores the transitions between non-conscious processing and conscious processing in a conscious subject. Many experiments in healthy controls and in various neurological disorders such as blindsight, neglect, visual hallucinations and agnosias can be accounted for by GW theory [34]. We could recently demonstrate that conscious access correlates with four neural signatures: 1) sustained voltage changes over distributed regions, particularly in prefrontal cortex, 2) large and sustained increases in spectral power in the gamma band, 3) increases in long-distance phase synchrony in the beta range, and 4) increases in long-range Granger causality [22].

On the other hand 'intransitive consciousness' refers to the mere existence of a functional conscious GW, – irrespective of its particular content –, in contrast with non conscious states such as deep sleep, comatose, general anesthesia, vegetative state, and generalized or complex partial seizures with AOC. Crucially, GW theory predicts that transitions between such non conscious states and conscious mode of processing follow non-linear threshold functions. More precisely, GW predicts that in response to progressive linear neuromodulatory increases (or decreases) of global cortical activation by the Ascending Reticular Activating System, long-distance cortico-cortical coherence would show a non-linear 'all-or-none' mode of activation (or deactivation).

These ideas were recently implemented in a computational model of GW network [14] in which ascending brain stem nuclei send globally depolarizing neuromodulatory signals to a thalamic and cortical hierarchy. Simulations confirmed that progressive increase in spontaneous firing of cortico-thalamic structures as a function of neuromodulator release evolves into what is known in dynamical systems theory as a Hopf bifurcation: spontaneous firing increases continuously in intensity, but high-frequency oscillations appear suddenly in the gamma band (20–80 Hz).

Taken together, these results strongly suggest that long-distance cortico-cortical synchronization in the beta and gamma band play a fundamental and causal role in conscious processing and top-down [10,11,21, 26].

The exploration of AOC contemporary to seizures can provide precious specifications of the range of neural coherence associated with conscious processing. In particular, excess of coherences are predicted to be a cause of conscious impairment, by reducing GW complexity and differentiation to dramatically poor values [37].

## 3. Alteration of Consciousness in Epilepsy: A summary of putative mechanisms

Temporal lobe seizures are the most frequent cause of partial seizures with AOC. Seizures in TLE are characterized by epileptic discharges originating from one or several regions of the temporal lobe (often from the

Table 1
The Consciousness Seizure Scale (CSS)

| Criteria | Assessment of the criteria |
|---|---|
| 1. Unresponsiveness (0 or 1). | The patient does not execute simple verbal commands (ex: "clap your hands", "open the mouth", "close your eyes"). |
| 2. No visual attention (0 or 1). | The patient presents no adequate visual response to external stimuli (ex: the patient does not look at the examiner during examination). |
| 3. No interaction with the examiner (0 or 1). | The patient does not present any signs (other than visual attention) of response to the examiner. |
| 4. No consciousness of the seizure (0 or 1). | The patient does not report to be in seizure state at any time of the seizure course (ex: he/she does not call the examiner at the beginning of the seizure). |
| 5. Inappropriate behavior (0 or 1). | The patient presents with an automatic, uninhibited behavior or an unreactive state. |
| 6. Post ictal amnesia (0 or 1). | The patient does not remember his/her seizure. |
| 7. Amnesia of the seizure events (0 or 1). | The patient does not remember the events that have occurred during the seizure. |
| 8. Global appreciation of consciousness by an experienced physician (0,1 or 2). | 0. No alteration 1.Middle alteration 2. Complete alteration . |

mesial temporal regions) and propagating through an interconnected network within both cortical and subcortical structures [27]. Video-EEG recordings have revealed that $\sim$ 60%–80% of patients suffering from TLE have AOC during their seizures [32]. AOC in TLE has been the subject of several studies initially based on intracerebral recordings [24,33]. The most frequent criteria for defining AOC is the absence of interaction with the examiner during seizure (loss of contact). One of the major limits of such studies is indeed the lack of accepted tool to measure AOC during an epileptic seizure. The clinical appreciation of AOC is a quite elusive concept in epileptology related to its subjective nature [24,30]. An exterior description cannot directly account for the subjective experience of the patient during his seizure. In general, clinical studies rely on indirect criteria for the measure of consciousness. More recent studies have proposed 2 to 4 criteria to estimate consciousness during seizures [6,7,29,31]. Recently we proposed a 8 criteria scale in order to capture the different elements for a more objective evaluation of the AOC during seizures (Table 1) [1].

Different hypothesis have been proposed to explain how those seizures could impair consciousness [20]. Intracerebral EEG recordings studies have suggested that alteration of consciousness could be related to the spread of epileptic discharge to cortical structures contralateral to the origin of the seizures and could be more frequent in seizures affecting the dominant hemisphere [31]. Bilateral involvement of the temporal lobe is a classical factor explaining the loss of memory but it has been shown that AOC may occur during apparently unilateral TLE seizures [24,33].

Metabolic neuroimaging studies using ictal SPECT in order to study the increase of cerebral blood flow (CBF) in regions affected by seizures have suggested that secondary involvement of thalamus and/or subcortical structures during seizures plays a determinant role in the impairment of consciousness [6,29].

In comparison with seizures not associated with AOC ("simple partial seizures"), seizures with AOC ("complex partial seizures") are associated with significant bilateral increase in CBF in the thalamic nuclei.

The involvement of thalamus during TLE seizures was directly shown by direct intracerebral recordings [27,35]. In one study, the degree of thalamic involvement was correlated to the presence of AOC during seizures [27].

According to SPECT and EEG approaches, Blumenfeld an colleagues also suggested that AOC occurs when the associative cortices are secondarily impaired [6,7] and proposed the "network inhibition" hypothesis. In this scenario, slow electrical activity over the associative cortices disrupt the normal functioning of cortices involved in normal attentional/conscious networks and create a situation close to the sleep slowing of the cortical activity. The "deactivation" of cortical structures could depend on subcortical driving under the influence of epileptic discharge.

## 4. Alteration of GWS functioning during TLE seizures

In line with these findings, we postulated that inadequate synchronization between subcortical and cortical modules constituting the GW could constitute a core mechanism of AOC in TLE.

It has been known for a long time that epileptic phenomena are associated with dramatic changes in brain

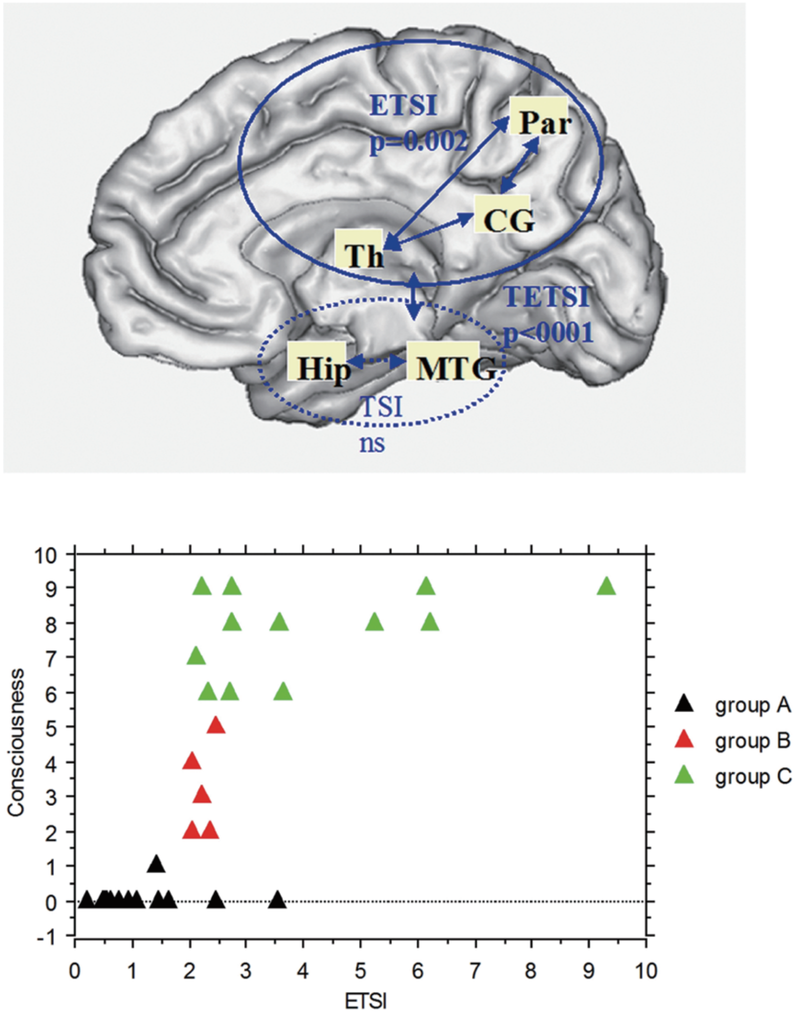

Fig. 2. Upper Part: Schematic spatial representation of three synchrony indices and their statistical variations by comparing group C and group A values during seizures. The temporal lobe synchrony index (TSI) represents the average of the $h^2$ values between signals from three studied temporal lobe structures (Hippocampus, Neocortex and Entorhinal cortex); the extra-temporal lobe synchrony index (ETSI) between three extra-temporal lobe regions (posterior cingulated, parietal cortex, thalamus) and the temporal/extra-temporal synchrony index (TETSI) between the three temporal and the three extra-temporal lobe regions. Seizures with AOC (group C) are characterized by significant higher synchrony indices in the extra-temporal interactions. Lower part: Relationship between ETSI values and the loss of consciousness estimated by the "seizure consciousness scale" (SCS). This relation follows a sigmoide curve suggesting a non-linear bistable function for cousciousness. (adapted from [1]).

synchrony mechanisms [9] and subsequent studies have shown that seizures in humans are associated with the abnormal synchronization of distant structures [4,5,25, 27]. This synchronization can be quantified by measuring the interdependencies between signals recorded in different brain regions involved in the epileptic seizures when invasive (depth electrodes) recordings are required (Fig. 1). Numerous methods were proposed over the past decades, often categorized according to their ability to assess the linear (coherence, linear regression analysis. . . ) or nonlinear (mutual information, nonlinear regression analysis, similarity between state-space trajectories reconstructed from observed signals) properties of the relationship [38].

Using these methods, it is therefore possible to study functional couplings between several brain regions involved or not during seizure. Among these, the so-called nonlinear regression analysis provides a parameter, referred to as the nonlinear correlation coefficient $h^2$, which takes values in [0, 1] and that has been particularly used to study epileptic seizures. Low values of $h^2$ denote that two signals X and Y under analysis

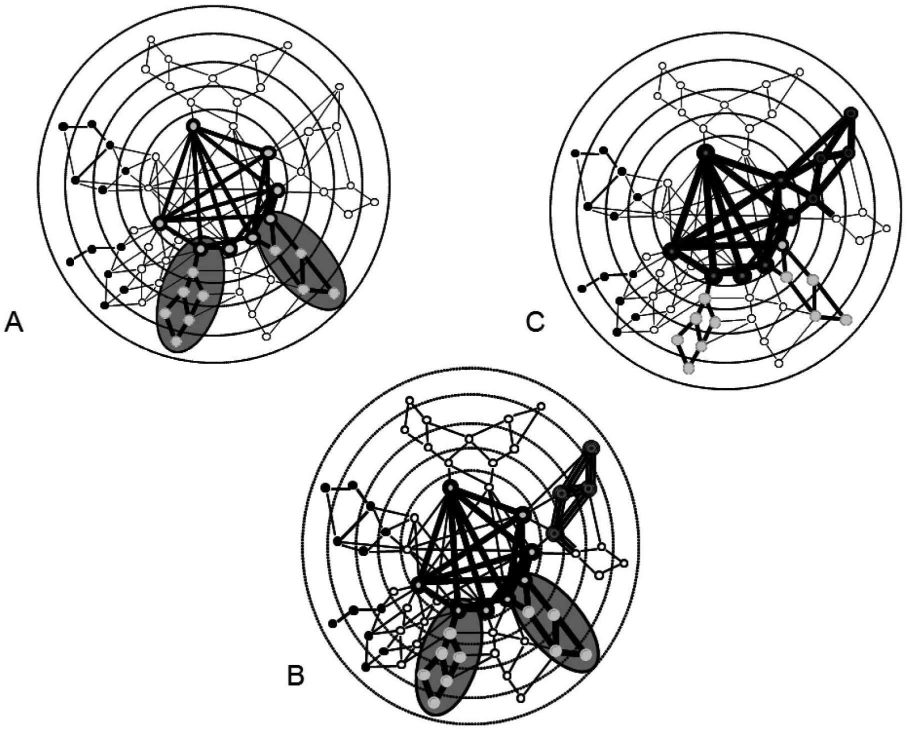

Fig. 3. Global Workspace (GW) theory of Consciousness and its alteration during seizures with consciousness alterations. A: Normal functioning in an awake subject. Some peripheral modules are engaged in cognitive processes (black circles). Two of them (large grey circles) are transiently engaged in the GW by transient long distance synchrony (thick black bars) linking associative cortices and subcortical structures. B: Seizure without alteration of consciousness (AOC) or period of onset in complex partial seizures. Seizure starts in a module altering local synchrony (red circles) and does not (yet) affect the functioning of GW. C: Seizure with phase of AOC. An excessive and sustained synchrony between GW core structures and the affected neural processor prevents the minimal level of GW complexity and information differentiation to be reached. As an immediate consequence, the GW is no more functional and consciousness disappears.

are independent. On the other hand, high values of $h^2$ mean that the second signal Y may be explained by a transformation (possibly nonlinear) of the first signal X (i.e. both signals are dependent).

Several studies have shown that TLE seizures are characterized by specific increase of neural synchrony that may largely involve regions distant from the sites of origin of the seizures [27]. Until recently however, the relationship between increased synchrony and AOC was unknown, even if prediction were made that increased synchrony could be associated with AOC [37].

This prompted us to investigate the relationship between altered synchrony in TLE seizures and AOC in patients having intracerebral recordings of cortical and subcortical structures as part of their presurgical evaluation [1].

In twelve patients, we have first quantified the AOC from video-SEEG recordings using the 8 criteria scale (CSS). Seizures were classified into three groups according to the degree of AOC: group A: no AOC, Group B: intermediate AOC; group C: complete AOC. We particularly studied the group C in comparison with group A.

The interdependencies between bipolar intracerebral EEG signals (derived from two contiguous leads of the same electrode) were estimated using non linear regression method and included three regions of the temporal lobe (two mesial regions: hippocampus (Hip) and entorhinal cortex (CE) and one lateral region: middle temporal gyrus (MTG)) and three regions outside the temporal lobe: thalamus (Th), lateral parietal cortex (P) and posterior cingulate gyrus (CG).

In comparison with the preictal ("background") period, the groups did not differed in term of neural synchrony within the temporal lobe at seizure onset. In contrast, a clear separation appeared between groups A and C, as only group C (with complete AOC) displayed marked enhanced $h^2$ values, particularly pronounced for interactions outside the temporal lobe (see example in Figs 1 and 2) suggesting the specific involvement of extra-temporal structures increased synchrony in AOC. A crucial result was to demonstrate that the degree of

AOC as measured by the SCS scale significantly correlated with the degree of thalamo-cortical and cortico-cortical synchrony of structures outside the temporal lobe (Fig. 2). The transition between consciousness and loss of consciousness as a function of synchronization followed a sharp sigmoid curve, suggesting a bi-stable system. Group B (intermediate AOC) values were spread in the narrow transition phase of the curve between the low and high $h^2$ values. Seizures without and with AOC recorded in the same patients distributed in the low and high $h^2$ values, respectively, further suggesting that increased cooperation outside temporal lobe regions underlies AOC.

In such a bi-stable system, intermediate conscious states are unstable, as reflected by the narrow transition zone between consciousness and LOC. This finding is consistent with a crucial prediction of the GW that consists of the non-linear property of conscious processing. According to the massive interconnectivity prevailing between the neural structures composing the GW, conscious processing is supposed to occur according to an "all or none" process [18,19,36].

Finally, we propose that during seizures with loss of consciousness, information cannot be processed within the GW because structures that are the most important for its activity are over-synchronized (in time and space). In contrast, seizures without loss of consciousness disturb the GW to a lesser degree, permitting at least partial functioning of long distance cortico-cortical connections between modules and the access to consciousness. This scenario is illustrated in Fig. 3.

## 5. Conclusions

Loss of consciousness is a frequent and dramatic aspect of partial seizures. In temporal lobe seizures it is linked to both cortical and subcortical mechanisms, and particularly to excessive synchrony between thalamus and associative cortices. In the future, new strategies aiming at preventing this excessive synchronization could dramatically improve patient's life and safety, even if seizures themselves are not suppressed.

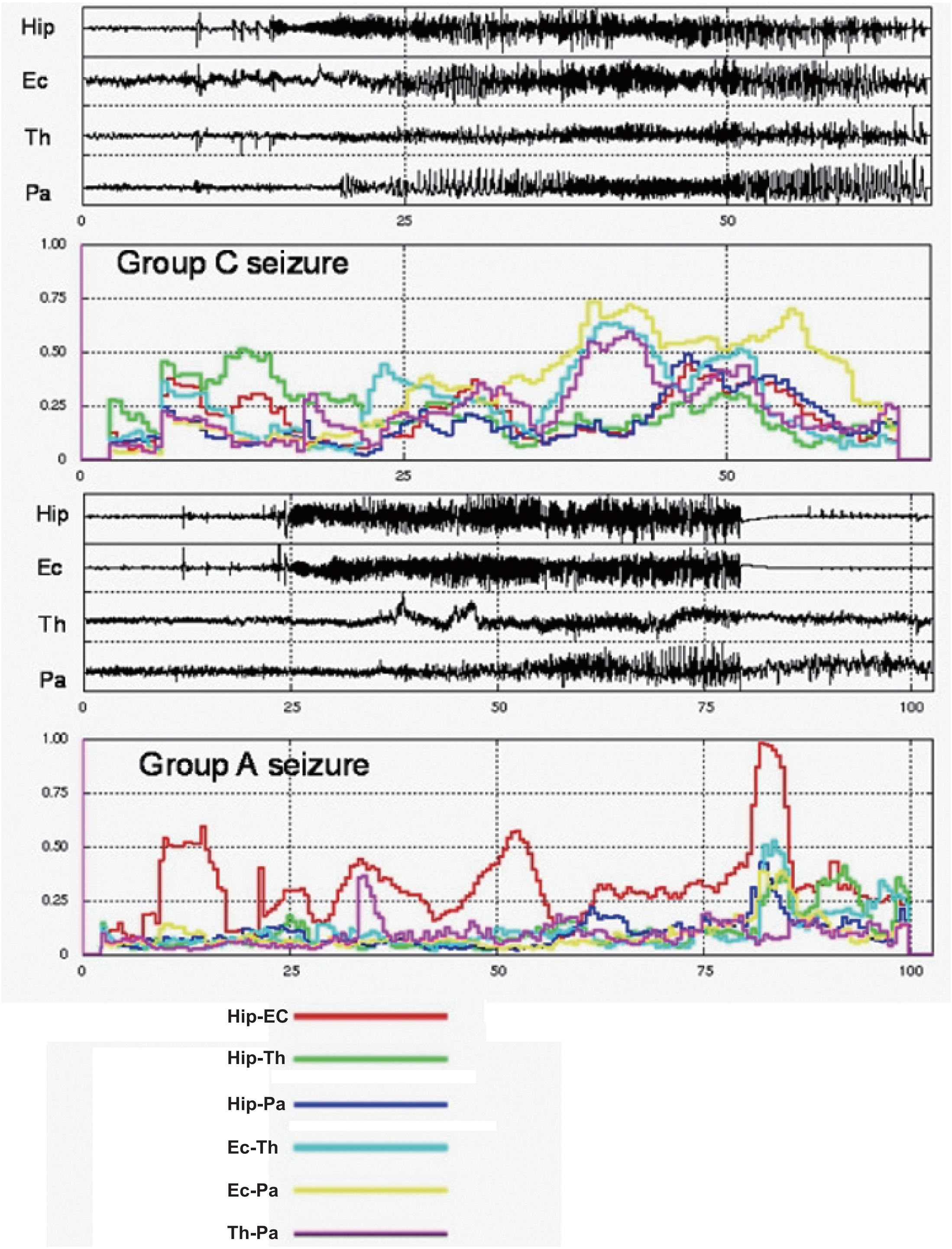

Fig. 1. Two exemples of seizure with AOC (group C) and without AOC (group A). Intracerebral recordings are obtained from multiple contacts electrodes placed according to Talairach's stereotactic method. Four regions are shown: Hip: Anterior Hippocampus, EC: entorhinal cortex; Th: Thalamus (pulvinar) and Pa: posterior parietal cortex. Seizures started in the medial temporal lobe before affecting thalamus and parietal cortex. In each case, the estimation of interdependencies used non linear regression ($h^2$) between pair wise signals. Increase in $h^2$ values is particularly marked for seizure from group C affecting not only the mesial temporal interactions (EC-Hip, red line) but also the other interactions represented. Seizure from group A is mainly characterized by increase in $h^2$ values between entorhinal cortex and hippocampus (red line).(adapted from [1]).
